# Association between Hemagglutinin Stem-Reactive Antibodies and Influenza A/H1N1 Virus Infection during the 2009 Pandemic

**DOI:** 10.1128/JVI.00093-16

**Published:** 2016-06-24

**Authors:** Le Nguyen Minh Hoa, Le Quynh Mai, Juliet E. Bryant, Pham Quang Thai, Nguyen Le Khanh Hang, Nguyen Thi Thu Yen, Tran Nhu Duong, Dang Dinh Thoang, Peter Horby, Heiman F. L. Werheim, Annette Fox

**Affiliations:** a Oxford University Clinical Research Unit and Wellcome Trust Major Overseas Programme, Hanoi, Vietnam; b National Institute of Hygiene and Epidemiology, Hanoi, Vietnam; c Center for Tropical Medicine, Nuffield Department of Clinical Medicine, University of Oxford, Oxford, United Kingdom; d Ha Nam Centre for Preventive Medicine, Ha Nam, Vietnam; e Department of Medical Microbiology, Radboudumc, Nijmegen, Netherlands; f The University of Melbourne, Peter Doherty Institute for Infection and Immunity, Department of Microbiology and Immunology, Parkville, Victoria, Australia; St. Jude Children's Research Hospital

## Abstract

The discovery of influenza virus broadly neutralizing (BrN) antibodies prompted efforts to develop universal vaccines. Influenza virus stem-reactive (SR) broadly neutralizing antibodies have been detected by screening antibody phage display libraries. However, studies of SR BrN antibodies in human serum, and their association with natural infection, are limited. To address this, pre- and postpandemic sera from a prospective community cohort study in Vietnam were assessed for antibodies that inhibit SR BrN monoclonal antibody (MAb) (C179) binding to H1N1 pandemic 2009 virus (H1N1pdm09). Of 270 households, 33 with at least one confirmed H1N1pdm09 illness or at least two seroconverters were included. The included households comprised 71 infected and 41 noninfected participants. Sera were tested as 2-fold dilutions between 1:5 and 1:40. Fifty percent C179 inhibition (IC_50_) titers did not exceed 10, although both IC_50_ titers and percent C179 inhibition by sera diluted 1:5 or 1:10 correlated with hemagglutination inhibition (HI) and microneutralization (MN) titers (all *P* < 0.001). Thirteen (12%) participants had detectable prepandemic IC_50_ titers, but only one reached a titer of 10. This proportion increased to 44% after the pandemic, when 39 participants had a titer of 10, and 67% of infected compared to 44% of noninfected had detectable IC_50_ titers (*P* < 0.001). The low levels of SR antibodies in prepandemic sera were not associated with subsequent H1N1pdm09 infection (*P* = 0.241), and the higher levels induced by H1N1pdm09 infection returned to prepandemic levels within 2 years. The findings indicate that natural infection induces only low titers of SR antibodies that are not sustained.

**IMPORTANCE** Universal influenza vaccines could have substantial health and economic benefits. The focus of universal vaccine research has been to induce antibodies that prevent infection by diverse influenza virus strains. These so-called broadly neutralizing antibodies are readily detected in mice and ferrets after infection with a series of distinct influenza virus strains. The 2009 H1N1 pandemic provided an opportunity to investigate whether infection with a novel strain induced broadly neutralizing antibodies in humans. We found that broadly neutralizing antibodies were induced, but levels were low and poorly maintained. This could represent an obstacle for universal vaccine development and warrants further investigation.

## INTRODUCTION

The ability of variant influenza strains to repeatedly infect humans fueled speculation that broadly neutralizing (BrN) antibodies are lacking (1). However, influenza virus BrN antibodies have been identified by screening cloned B cells or phage display libraries, albeit rarely (2–5). Animal studies directly demonstrate that influenza virus BrN antibodies can be elicited, and this has driven efforts to develop universal vaccines (6). Most of the BrN antibodies identified bind conserved epitopes in the hemagglutinin (HA) stem that are comprised largely of the HA2 subunit and prevent pH-dependent conformational changes required for fusion of viral and cellular membranes (2, 4, 7, 8). The crystal structures for several stem-reactive (SR) BrN monoclonal antibodies (MAbs) in complex with HA indicate that they target a similar epitope, which is a highly conserved pocket containing the fusion peptide (FP) (4, 9). FP is conserved among H1N1 strains and other closely related HA subtypes (4). C179 is an SR BrN mouse MAb (4, 10) that interacts with surface amino acids of FP (i.e., HA2 positions 18 to 21), together with 15 other HA2 and HA1 amino acids within the stem (9). The immunodominance of the globular head of HA (11) and inaccessibility of the HA stem (7) may preclude induction of fusion-inhibiting antibodies (6).

Few studies have assessed SR BrN antibody levels in human serum, the factors associated with their detection, or whether detection is associated with protection. In previous studies involving the Ha Nam community influenza cohort in Vietnam, we found that increasing age and prior seasonal H1N1 infection were associated with protection against pandemic H1N1 infection in the absence of detecting hemagglutination-inhibiting (HI) antibodies (12). Therefore, in the current study, we examined whether HA SR antibodies could be detected in serum samples spanning the pandemic, and whether detection was associated with reverse transcription-PCR (RT-PCR)-confirmed or serologically defined infection. HA SR antibodies were detected by an enzyme-linked immunosorbent assay (ELISA) measuring inhibition of C179 MAb binding to A/Cal/07/09-like (H1N1pdm09) virus. This ELISA may detect antibodies that can neutralize virus by binding directly to the C179 MAb epitope, but it could also detect antibodies that bind nearby, nonneutralizing epitopes and inhibit C179 binding via steric hindrance. Therefore, we examined avian H6 virus-neutralizing activity of sera. Avian H6 viruses and H1N1pdm09 have distinct HA globular heads, but C179 can bind both and the epitope is quite conserved (9).

## MATERIALS AND METHODS

### Study design.

This study examined pre- and postexposure sera from people with and without subsequent infection to investigate associations between antibodies and protection or infection. The study utilized stored sera from the Ha Nam community influenza cohort, which has been described in detail previously (13). The cohort was established in 2007 and comprised 270 houses and 945 participants at the time of the 2009 pandemic. To minimize confounding effects of hemagglutination-inhibiting (HI) antibodies, the current investigation focused on the 2009 H1N1 pandemic because HI antibodies against the pandemic strain were rarely detected in prepandemic sera from cohort participants (12). Participants were considered to have been infected if they had an RT-PCR-confirmed influenza-like-illness (ILI) or seroconverted in an HI assay. Participants with ILI, defined as fever (oral temperature above 38°C) with cough and/or sore throat, were identified by continuous active surveillance. Respiratory swabs were collected by health care workers, and RT-PCR was performed to detect influenza virus as described previously (14). HI assays were performed on paired pre- and postpandemic sera to detect seroconversion, defined as a titer rise of at least 4-fold (12). To increase the likelihood that noninfected participants were exposed, and therefore of detecting antibodies associated with protection, households with at least one confirmed H1N1pdm09 illness or at least two people who seroconverted without ILI being detected were selected. The research was approved by the institutional review boards of the National Institute of Hygiene and Epidemiology, Vietnam, and the Oxford Tropical Research Ethics Committee, University of Oxford, United Kingdom. All participants provided written informed consent.

### C179 inhibition ELISA to detect HA stem-reactive (SR) antibodies.

**FIG 1:**
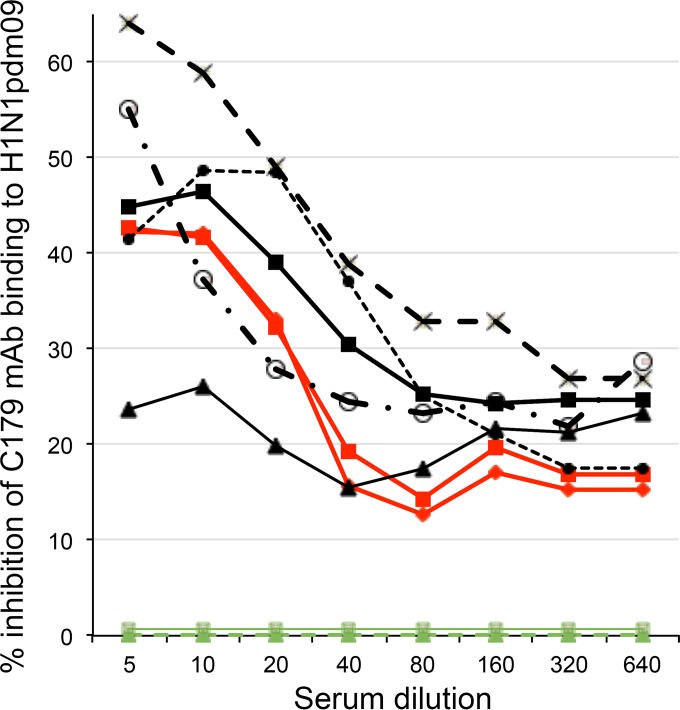
Inhibition of C179 MAb binding to H1N1pdm09 by serial dilutions of first-infection ferret antisera, raised against H1N1 A/Cal/07/09-like virus (red symbols) or H3N2 A/Vic/361/2011-like and A/Texas/50/2012-like viruses (green symbols), or postpandemic sera from H1N1pdm09-infected cohort participants (black symbols).

ELISA was used to detect serum antibodies that inhibit fusion-blocking MAb clone C179 (catalog no. M145; Clontech Laboratories) binding to an A/Cal/07/09-like (H1N1pdm09) virus. Ninety-six-well plates (catalog no. 439454; Nunc) were coated overnight at 4**°**C with H1N1pdm09 virus (supplied by the Victorian Infectious Diseases Reference Lab [VIDRL], Melbourne, Australia, as part of the 2012 influenza virus typing kit). Twofold virus dilutions in carbonate-bicarbonate buffer, pH 9.6 (catalog no. C3401; Sigma), were compared, and 80 HA units was chosen as the optimal dilution for C179 binding. Virus-coated plates were blocked with 3% bovine serum albumin (BSA) in PBS-Tween and then incubated with serum dilutions for 2 h at 37°C before addition of 2 μg/ml of C179 MAb and incubation for a further 1 h. C179 binding was detected with goat anti-mouse IgG conjugated to horseradish peroxidase (catalog no. 074-1802; Kirkegaard & Perry Laboratories) and then *o*-phenylenediamine dihydrochloride (OPD; catalog no. P8287; Sigma). Each plate contained four virus control wells, as well as four background subtraction wells coated with BSA. Background absorbance was subtracted before calculating the percentage inhibition of C179 binding as follows: 100 − [(OD_490_ with serum/OD_490_ of virus control) × 100], where OD_490_ is optical density at 490 nm. Comparison of C179 MAb binding to plates coated with A/Cal/07/2009-like H1N1 versus that with A/Perth/16/09-like H3N2 virus (VIDRL) verified that the assay detects C179 binding to the former (*A*_490_ > 0.5) but not the latter (*A*_490_ < 0.07). Reference sera (first-infection ferret sera for influenza typing, supplied by the VIDRL) were tested as a potential positive control and a specificity control for group 1 versus group 2 FP-reactive antibodies. A/Cal/07/09-specific reference sera, but not A/H3N2 (A/Victoria/361/2011 or A/Texas/50/2012)-specific reference sera, inhibited C179 binding to A/Cal/07/09-like virus (Fig. 1). C179 binding inhibition was less for A/Cal/07/09-specific reference sera than for participant postinfection sera tested in the same assay, although the HI titer was higher for A/Cal/07/09-specific reference sera (1,280) than for these participant sera (40 to 160), indicating that HI antibodies do not inhibit C179 binding.

### HI assay, MN assay, and PRNT.

Paired sera were tested in the HI assay as previously described (13). The microneutralization (MN) assay was performed according to the World Health Organization protocol (http://www.who.int/influenza/gisrs_laboratory/2010_12_06_serological_diagnosis_of_influenza_by_microneutralization_assay.pdf). Briefly, 100× 50% tissue culture infective dose (TCID_50_) of virus (A/Cal/07/09-like isolate propagated in MDCK cells) was mixed in equal volume with 2-fold serial dilutions of serum and incubated for 2 h at 37°C. MDCK cells (1.5 × 10^4^/well) were added to the plates, followed by incubation at 37°C for 20 h. Cells were fixed, and viral antigen was detected by indirect ELISA with a primary mouse MAb against influenza A virus nucleoprotein (catalog no. sc-101352; Santa Cruz) followed by goat anti-mouse IgG conjugated to horseradish peroxidase (as mentioned above) and then OPD (as mentioned above). A cutoff, or OD value at which 50% of the MDCK cells were infected, was determined using the following equation: cutoff = (average OD_490_ of virus control wells − average OD_490_ of cell control wells)/2. All serum dilutions with OD_490_ values below or equal to the cutoff were considered positive for neutralization activity. The reciprocal of the highest positive serum dilution was inferred to be the neutralizing antibody titer for that serum sample. PRNT was performed according to the protocol of Margine et al. (15), in which matched serum dilutions are added to the agar overlay. Modifications of this protocol included the use of influenza virus nucleoprotein-reactive antibody to detect virus or plaques, as described above for the MN assay, and the use of 96-well plates and an automated reader to count plaques as described by Zielinska et al. (16). The A/H6 virus used was isolated from a duck from southern Vietnam in 2012, propagated in embryonated hen's eggs, and then passaged six times in MDCK cells. The HA gene of this virus was sequenced, and amino acids used at C179 contact positions were compared with those of viruses that have been shown to bind to C179 (9). C179 contact positions were identical between the H6 virus used and an H6N2 virus that binds C179 with intermediate affinity (*K_d_* [dissociation constant] = 40 nM) (9), with the exception of a conservative arginine-to-lysine substitution at HA2 residue 38 (Table 1). However, six C179 binding residues differed between H6 and H1N1pdm09, of which three were nonconservative substitutions. Positive control serum (rabbit hyperimmune serum against A/duck/Shantou/5540/01 [H6N2] [17]) was provided by Richard Webby (St. Judes) and had a titer of 160 against the duck virus.

**TABLE 1:**
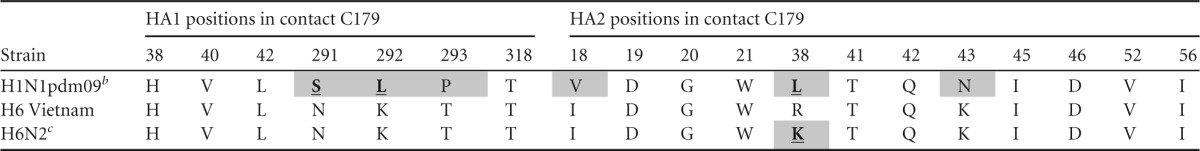
Comparison of amino acids at C179 MAb contact positions of the Vietnam duck H6 virus used for PRNT versus previously characterized H6N2 and H1N1 viruses^*a*^

a C179 contact positions are based on studies of Dreyfus et al. (9). Positions that differ are shaded. Bold underlined letters indicate nonconservative substitutions.

^b^ A/California/04/2009 (9).

^c^ A/turkey/Massachusetts/3740/1965 (9).

### Statistical analysis.

The Mann-Whitney test was used to compare antibody levels or titers and participant ages for infected versus noninfected or pre- versus postpandemic groups. Proportions were compared using the chi-square test. Pearson and Spearman correlation coefficients were used to indicate the correlation between two different antibody assays. Binary logistic regression was also used to assess the effect of the prepandemic FP-reactive antibody level on infection during the first pandemic wave. Generalized estimating equations were used to account for household clustering. Statistical analyses were performed with SPSS 19 statistical software.

## RESULTS

### Analysis of included participants.

**FIG 2:**
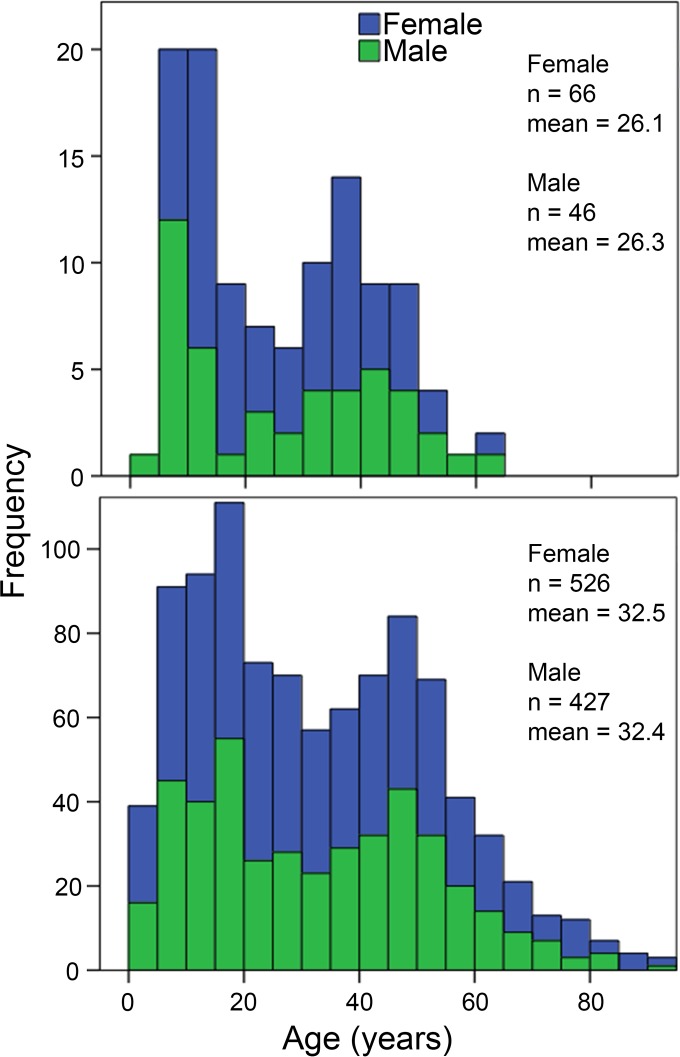
Age and gender distribution of participants selected for analysis of HA stem-reactive antibodies (top) compared to all cohort participants (bottom).

Thirty-three of 270 households in the Ha Nam cohort contained at least one participant with H1N1pdm09 illness (8 houses) or at least two H1N1pdm09 seroconverters (15 houses) or both (10 houses) and were selected for analysis of SR antibodies in serum. Of 129 participants residing in these houses, 112 provided prepandemic sera in June 2009, and 107 also provided postpandemic sera in April 2010. Seventy-one of the included participants were infected, including 23 with ILI, and 41 were not infected during the 2009 H1N1 pandemic. The age and gender distribution of selected households was similar to that of all households in the cohort (Fig. 2). The median (interquartile range [IQR]) age was 15.8 (9.6 to 33.3) years for infected participants, compared to 37.3 (27.1 to 45.8) years for noninfected participants. This difference was significant (*P* < 0.001), consistent with a previous analysis of all households in the cohort (12).

### Detection of influenza virus HA SR antibodies in pre- and postpandemic sera via C179 inhibition ELISA.

**FIG 3:**
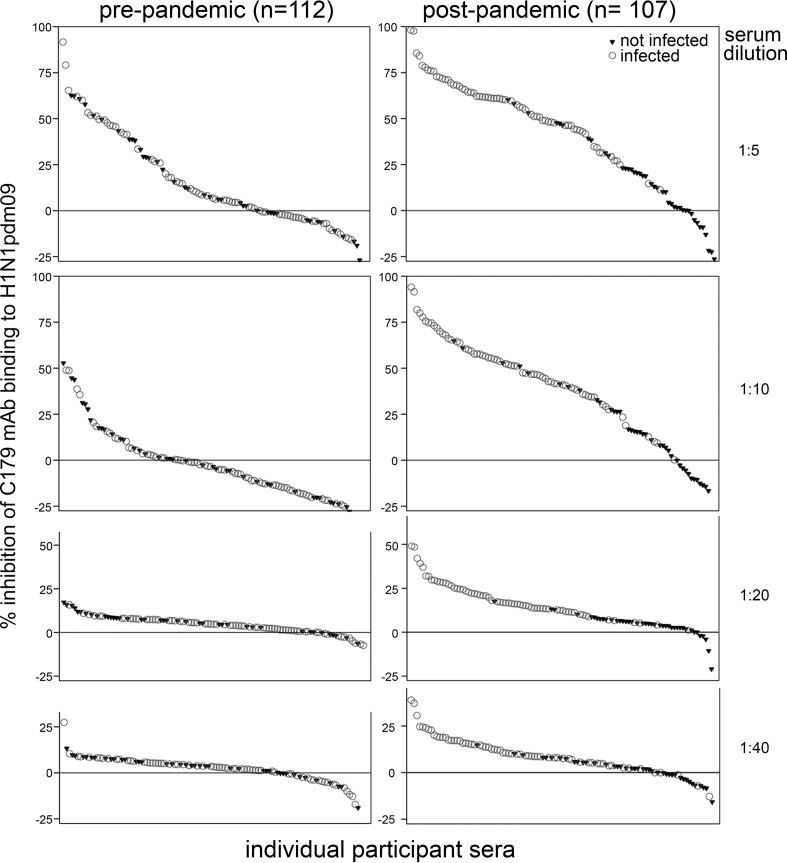
SR antibody detection in pre- and postpandemic sera at different dilutions. SR antibody levels in sera diluted 1:5, 1:10, 1:20, or 1:40 were determined by ELISA to detect inhibition of C179 binding to H1N1pdm09 virus. Each symbol represents an individual's serum and indicates H1N1pdm09 infection status.

Sera were assessed over 2-fold dilutions from 1:5 to 1:40 to detect antibodies that inhibit binding of FP-reactive MAb (C179) to H1N1pdm09 virus. These antibodies are also referred to here as SR antibodies. Fifty percent inhibitory (IC_50_) titers were low and did not exceed 10 in either pre- or postpandemic sera (Fig. 3). Only 12% of prepandemic sera had detectable IC_50_ titers (Table 2), one had an IC_50_ titer of 10, and the remainder had titers of 5, whereas 34% had detectable IC_20_ titers (Table 2). The proportion with detectable IC_50_ titers rose to 44% after the 2009 H1N1 pandemic and was significantly higher among infected (67%) than among noninfected (10%; *P* < 0.001) participants. The proportion of infected participants who had detectable postpandemic IC_20_ titers approached 100% (Table 2). The percent inhibition of C179 binding differed only marginally between sera diluted 1:5 and 1:10 (Fig. 3). Therefore, results are also presented as percent inhibition of C179 binding by sera diluted 1:5 and 1:10 to yield greater discrimination between sera than indicated by titer.

**TABLE 2:** Proportions of participants with detectable 20% or 50% C179 binding inhibition titers

Titer	No. (%) of sera			
	Prepandemic (*n* = 112)	Postpandemic (*n* = 107)	Postpandemic, infected (*n* = 66)	Postpandemic, not infected (*n* = 41)
IC_20_				
5	26 (23)	6 (6)	3 (5)	3 (5)
10	12 (11)	75 (70)	61 (92)	14 (34)
IC_50_				
5	12 (11)	9 (8)	9 (14)	0 (0)
10	1 (1)	39 (36)	35 (53)	4 (10)

### Relationship between SR and neutralizing antibody titers, and effect of time since infection.

**FIG 4:**
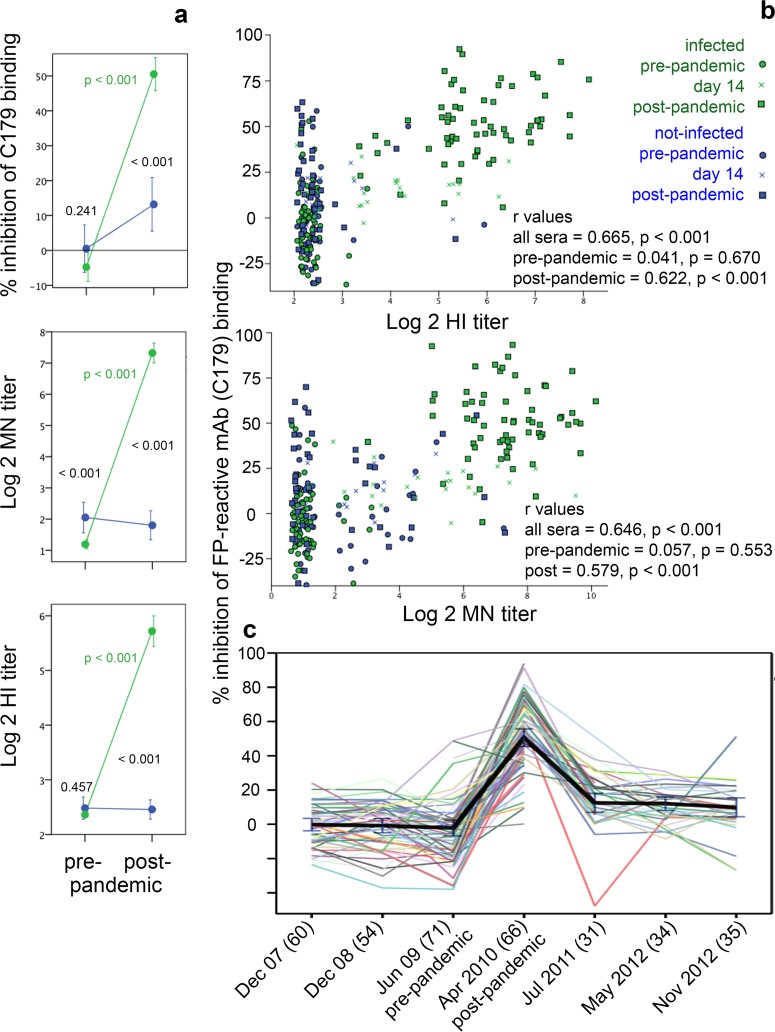
H1N1pdm09-reactive antibody detection via ELISA versus HI and MN assay. Panels a and b show antibody levels for infected (green; *n* = 71) versus noninfected (blue; *n* = 41) participants. Mean levels (±95% confidence intervals [CIs]) for each type of antibody in pre- and postpandemic sera are shown in panel a. *P* values for the comparison of infected and noninfected participants at each time point are in black text, whereas those for the comparison of pre- and postpandemic levels among infected participants are in green text. Panel b shows correlations between C179 inhibition ELISA and MN titer for prepandemic (top) and postpandemic (bottom) sera. Pearson correlation coefficients are shown. Inferences were equivalent for Spearman correlations. Data points have been jittered (http://www.ats.ucla.edu/stat/spss/faq/jitter.htm) to facilitate visualization of results for multiple individuals with the same titers. Panel c shows levels of C179-inhibiting antibody over time for each infected participant (colored lines), with means (black line) and 95% CIs (error bars). Numbers of participants tested at each time point are shown in parentheses. Results are shown for C179 inhibition ELISA with sera diluted 1:10.

The percent C179 MAb inhibition by sera diluted 1:10 increased significantly upon infection, and levels in postpandemic sera were significantly higher among infected than among noninfected participants (Fig. 4a), further indicating that SR antibodies are induced by infection. Inhibition levels in prepandemic sera were similar in those who became infected or who remained uninfected (Fig. 4a) and were not associated with subsequent infection status during the first pandemic wave (*P* = 0.241). Inhibition levels in postpandemic sera diluted 1:10 correlated with MN titers (Fig. 4b). Similar results were obtained for correlations with HI assay (*r* = 0.579; *P* < 0.001), for sera diluted 1:5 (ELISA versus HI, *r* = 0.592; ELISA versus MN, *r* = 0.540), and for correlations with IC_50_ titers (all *P* < 0.001). However, correlations were poor and nonsignificant for sera collected prepandemic (Fig. 4b), when only 6 and 15 participants had detectable HI and MN titers, respectively. Analysis of longitudinal serum samples from infected participants revealed that C179 inhibition fell dramatically by July 2011, i.e., less than 2 years after infection (Fig. 4c), indicating that SR antibodies are poorly maintained.

### PRNT titers against H6 and H1N1pdm09 viruses in pre- and postpandemic sera.

**FIG 5:**
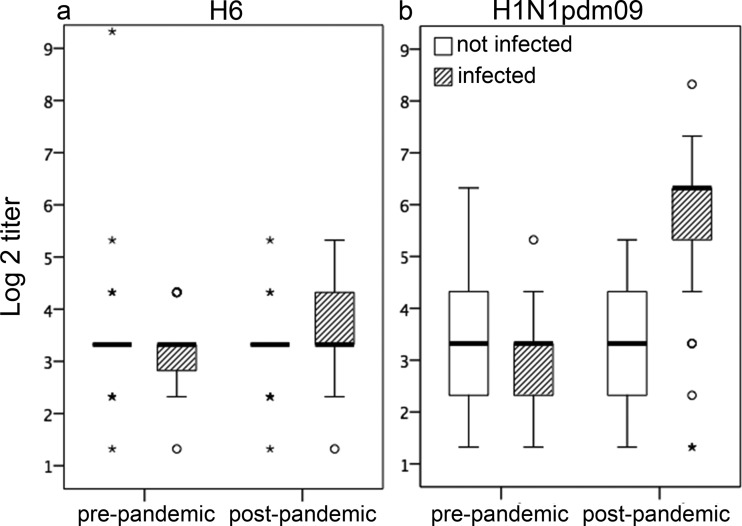
PRNT titers against H6 and H1N1pdm09 viruses. Pre- and postpandemic sera from 46 H1N1pdm09-infected and 31 noninfected participants were assessed in a PRNT with a duck H6 virus (a) and H1N1pdm09 virus (b). Log_2_ titers are summarized as box-and-whisker plots showing median, interquartile range, and range.

Avian H6 virus-neutralizing activity was assessed for sera from a subset of 31 noninfected and 46 infected participants. H6 PRNT titers were higher in postpandemic than in prepandemic sera from infected but not noninfected participants (Fig. 5a); however, the increase was small and not significant (*P* = 0.104). A total of 33.3% of infected participants had postpandemic H6 PRNT titers of 20 or more, compared to 9.7% of noninfected participants. H6 PRNT seroconversion was detected in only one participant, who was infected. PRNT titers against H1N1pdm09 rose among infected, but not noninfected, participants, as expected (Fig. 5b). H6 neutralizing antibodies could not be detected via the MN or HI assay (data not shown), indicating that the antibodies detected by the PRNT did not prevent virus attachment.

H6 PRNT and C179 inhibition ELISA titers were only weakly correlated using the Spearman nonparametric test (*r* = 0.177; *P* = 0.029). Correlations were stronger when analysis was limited to the infected group (*r* = 0.207; *P* = 0.050) or to postpandemic sera (*r* = 0.247; *P* = 0.031) but not as strong as correlations between H6 and H1N1pdm09 PRNT results (*r* = 0.307; *P* < 0.001) or between C179 inhibition and H1N1pdm09 PRNT titer (*r* = 0.438; *P* < 0.001).

## DISCUSSION

This study examined whether antibodies that react with the stem of H1N1pdm09 HA were associated with infection during the 2009 H1N1 pandemic. We examined the ability of sera to react with the HA stem by measuring inhibition of binding of C179 MAb, which is known to bind an epitope surrounding the FP of H1N1, but not H3N2, viruses (10). Stem-reactive (SR) antibody levels increased significantly upon H1N1pdm09 infection, and postpandemic titers correlated significantly with HI and MN titers. Correlation between SR and HI or MN assays was notably poor for prepandemic sera, which lacked HI activity against H1N1pdm09. SR antibodies could be detected in some prepandemic sera at low levels, consistent with substantial C179 epitope conservation between seasonal and pandemic H1N1 (9). These low SR antibody levels in prepandemic serum were not associated with protection against H1N1pdm09 infection, and postpandemic boosting was transient. Sera from one-third of participants who had been infected contained H6-neutralizing antibodies detectable by the PRNT but not by the MN or HI assay. Similarly, Pica et al. detected boosting of H6-reactive antibodies by the PRNT, but not by the HI or MN assay, after H1N1pdm09 infection, but they assessed purified IgG from pooled sera rather than individual sera (18).

The protective effect of fusion-inhibiting antibodies against challenge infection has been well established in animal vaccination and serial infection models (2, 4, 19, 20). The current study did not detect an association between protection and SR antibodies, but the levels in prepandemic sera were low and probably did not reach levels equivalent to those induced in animal models. Furthermore, SR antibody levels were only weakly correlated with H6 PRNT titers, which may indicate that C179-inhibiting antibodies bind nearby epitopes and have a steric effect on C179 binding but do not prevent conformational change and virus fusion. However, it is also possible that C179-inhibiting antibodies induced by H1N1 and H1N1pdm09 infection have poor reactivity against H6 because the epitope varies, a suggestion consistent with the stronger correlation between C179 inhibition and H1N1pdm09 PRNT titer.

Our findings that SR antibodies were detected at relatively high levels following infection with the novel H1N1pdm09 virus are consistent with a number of studies of antibody-secreting cells (ASC) induced following influenza virus infection or vaccination. ASC that make SR BrN antibodies are induced upon first exposure to antigenically novel viruses, such as H1N1pdm09 or H5N1, but rarely after exposure to seasonal influenza viruses, which favor ASC that react with strain-specific epitopes in the bulky head of HA (7, 8, 21). The increased detection of HA SR ASC following H5N1 vaccination was also accompanied by increased detection of FP-reactive antibodies in sera via inhibition ELISA (21). Andrews et al. recently confirmed that SR BrN antibody induction was not maintained after a second immunization with H1N1pdm09 vaccine, due to the immunodominance of the HA head and the presence of strain-specific neutralizing antibodies and memory B cells (22).

It is not known whether the higher levels of SR antibodies detected in response to novel strains would be sufficient for protection. Even so, boosted levels were not sustained, a finding of concern for the development of vaccines that target fusion-inhibiting epitopes to elicit broad protection. SR BrN antibodies are predominantly encoded by variable heavy (VH) chain genes, such as VH1-69, which encode long, compact complementarity-determining region 2 (CDR2) loops. These CDR2 loops enable binding to inaccessible hydrophobic epitopes such as FP (2, 4, 7, 8, 23). In addition, specific mutations in the VH CDR2 loop appear to be necessary for affinity maturation (23). VH1-69 and similar VH genes are also predominantly used by HIV and hepatitis C virus (HCV) BrN antibodies and by self-reactive antibodies associated with autoimmune diseases (24). Andrews et al. investigated the mechanism underlying the restricted VH gene usage by influenza virus SR BrN antibodies (22). Similar to HIV and HCV BrN antibodies, influenza virus SR BrN antibodies were able to bind multiple antigens such as double-stranded DNA (dsDNA), lipopolysaccharide (LPS), and insulin with low affinity via noncovalent, hydrophobic, and charge interactions. They also demonstrated that influenza virus SR BrN antibodies have reduced binding to whole virions but not HA protein compared to that of HA head-reactive antibodies, and this corresponded with reduced epitope association rates demonstrated by surface plasmon resonance (22). They therefore speculate that SR BrN antibodies use a restricted set of VH genes that can bind poorly accessible epitopes, albeit with low affinity, because SR BrN antibody epitopes are relatively inaccessible in whole virions (22). HIV BrN antibodies are particularly self-reactive or autoreactive, and therefore, B cell anergy could account for their rarity (25). Self-reactive B cells can escape anergy upon recognition of a foreign antigen by incorporating mutations that reduce self-reactivity while increasing foreign antigen affinity, but this may not be possible in the case of some broadly neutralizing antibody epitopes (26). Interestingly, CDR2 region mutations are critical for reducing self-reactivity (26).

The current study has several limitations that impede interpretation of the effects of SR antibodies on infection. As discussed above, prepandemic SR antibody levels were low, and the levels required for protection have not been determined. The low levels of SR antibodies also meant that there was little resolution between sera if IC_50_ titers were used, and another cutoff could be considered. The cutoff is also calculated based on the assumption that C179 binding can be fully inhibited, which did not occur. SR antibody detection could potentially be improved by using recombinant HA protein instead of whole virus (22), but this would not reflect the activity of serum antibodies against virus. Additionally, the ELISA to detect C179-inhibiting antibodies may not detect all antibodies that inhibit fusion. Multiple fusion-inhibiting MAbs have now been described, and some of the epitopes recognized have little overlap with the C179 epitope (27). Alternatively, the ELISA may detect antibodies that inhibit C179 binding but not fusion, as indicated by comparison with H6 PRNT. Nevertheless, the results suggest that SR antibodies were unlikely to account for the effects of age and prior seasonal H1N1 infection on pandemic H1N1 infection (12). It remains to be determined whether higher levels of SR antibodies protect humans against novel influenza A viruses. It will also be important to investigate whether loss of SR antibodies reflects autoreactivity and poses a potential obstacle for development of a universal vaccine.
